# The effects of IL-10 gene polymorphism on serum, and gingival crevicular fluid levels of IL-6 and IL-10 in chronic periodontitis

**DOI:** 10.1590/1678-7757-2017-0232

**Published:** 2018-02-15

**Authors:** Hulya Toker, Emine Pirim Gorgun, Ertan Mahir Korkmaz, Hatice Balci Yüce, Omer Poyraz

**Affiliations:** 1 Cumhuriyet University Cumhuriyet University Faculty of Dentistry Department of Periodontology Sivas Turkey Cumhuriyet University, Faculty of Dentistry, Department of Periodontology, Sivas, Turkey.; 2 Cumhuriyet University Cumhuriyet University Faculty of Sciences Department of Molecular Biology and Genetics Sivas Turkey Cumhuriyet University, Faculty of Sciences, Department of Molecular Biology and Genetics, Sivas, Turkey.; 3 Gaziosmanpasa University Gaziosmanpasa University Faculty of Dentistry Department of Periodontology Tokat Turkey Gaziosmanpasa University, Faculty of Dentistry, Department of Periodontology, Tokat, Turkey.; 4 Cumhuriyet University Cumhuriyet University Faculty of Medicine Department of Microbiology Sivas Turkey Cumhuriyet University, Faculty of Medicine, Department of Microbiology, Sivas, Turkey.

**Keywords:** Gingival crevicular fluid, Interleukin-10, Periodontal disease, Polymorphism

## Abstract

**Objective:**

Anti-inflammatory cytokines play a crucial role in periodontitis by inhibiting synthesis of pro-inflammatory cytokines. The purpose of this study was to evaluate the effect of interleukin-10 (-597) gene polymorphism and genotype distributions on chronic periodontitis (CP) development and IL-6 and IL-10 levels in gingival crevicular fluid (GCF) and serum before and after non-surgical periodontal treatment.

**Material and Methods:**

The study population consisted of 55 severe generalized CP patients as CP group and 50 healthy individuals as control group. Plaque index, gingival index, probing depth and clinical attachment level were recorded and GCF and blood samples were taken at both the baseline and the sixth week after non-surgical periodontal treatment. PCR-RFLP procedure was used for gene analyses and cytokine levels were measured via ELISA.

**Results:**

IL-10 genotype distribution was significantly different between CP and control groups (p=0.000, OR:7, 95%CI, 2.83-60.25). Clinical measurements significantly improved in the CP group after periodontal treatment (p<0.05). Periodontal treatment significantly decreased GCF IL-6 and IL-10 levels. No significant difference was found in clinical parameters between IL-10 AA and AC+CC genotypes at both the baseline and the sixth week (p>0.05). Sixth week GCF IL-10 levels were significantly lower in patients carrying IL-10 AC+CC genotype compared to the patients carrying IL-10 AA genotype (p<0.05). Serum IL-6 and IL-10 levels were lower in patients carrying the IL-10 AA genotype compared to patients with IL-10 AC+CC genotype, but the difference was not significant (p>0.05).

**Conclusion:**

IL-10 AA genotype carriers had lower IL-6 and IL-6/10 levels in serum; however, GCF IL-6/10 levels were similar in both genotypes. Within the limitations of our study, a possible association between IL-10(-597) gene polymorphism and CP might be considered.

## Introduction

Chronic periodontitis (CP) is the inflammatory and infectious disease of the soft and hard tissues around teeth. Etiopathogenesis of the disease involves many factors such as environmental and genetic factors^22^. Cytokines are the most important candidates for involvement of gene polymorphisms in CP development; possible associations between CP and certain gene polymorphisms were suggested^7^. The structure of a protein and/or its expression might change by polymorphism of the specific gene, thus, innate and adaptive immunity may be compromised^13^^,^^27^. Therefore, gene polymorphisms might have a deterministic importance in disease outcome.

One of the most important factors responsible for periodontal destruction is the upregulation of pro-inflammatory cytokines. Interleukin-6 (IL-6), as one of these pro-inflammatory cytokines, was reported to be an effective stimulator of osteoclast differentiation and bone resorption^21^. Along with CP, several inflammatory diseases were also associated with elevated levels of IL-6^12^^,^^17^. On the other hand, anti-inflammatory cytokines such as IL-10 can restore balance by inhibiting synthesis of pro-inflammatory cytokines (IL-1, IL-6, TNF-alpha) and stimulating protective antibody production^20^^,^^26^. For this reason, IL-10 gene polymorphism might contribute to periodontitis development.

The gene encoding human IL-10 is located on chromosome one (1q31-32). IL-10 three biallelic polymorphisms within the IL-10 promoter region, at positions -1087, -824, and -597 from the transcription initial site, have also been identified^23^. Although decreased IL-10 levels with IL-10 promotor gene polymorphism were reported, studies evaluating the relationship between this single nucleotide polymorphisms (SNPs) and CP reported conflicting results^3^^,^^8^^,^^11^^,^^20^^,^^23^.

Several researches on the clinical utility of cytokine genotyping for periodontal disease were conducted, but the genetic knowledge of periodontitis pathogenesis and effects on treatment results is still limited. Previously, we reported that IL-6 (-174) GG genotype caused an increase in GCF IL-10 levels without affecting periodontal treatment outcomes and we also found that SNPs in IL-6 (-174) and IL-10 (-597) were associated with generalized aggressive periodontitis (GAgP)^18^. However, there is no information regarding the ratio of inflammatory markers (IL-6/IL-10) in different genotype distribution of IL-10 gene in chronic periodontitis. Therefore, the aim of our study was to assess the IL-10 -597 SNPs in CP patients and to evaluate the effect of polymorphisms on non-surgical periodontal therapy and serum and GCF cytokine (IL-6 and IL-10) levels. Secondly, a possible association between IL-10 genotypes and local and systemic inflammatory status presented as the ratio of IL-6/IL-10 was investigated.

## Material and methods

### Study population

A total of 105 participants were involved in this study. CP group consisted of 55 chronic periodontitis patients (CP group) and control group consisted of 50 healthy volunteers (HC group). All participants were systemically healthy and never smoked. Ethical approval was provided by the Ethical Committee of Human Researches of Cumhuriyet University Faculty of Medicine, according to the Helsinki declarations (2012-02/035). All participants were informed and signed the informed consent form.

Exclusion criteria were: presence of conservative or prosthetic restorations in the anterior region, systemic disease, obesity, pregnancy or lactation, antibiotic or anti-inflammatory drug use within three months, requirement of antibiotic prophylaxis, infectious diseases such as hepatitis and HIV infection, chemotherapy, and periodontal treatment within six months.

Chronic periodontitis was diagnosed based on clinical examination. 30% of total teeth in CP patients had CAL values higher than 5 mm^1^. Periodontally healthy individuals had no alveolar bone loss in radiographs. Control group also had full mouth probing depth with less than 3 mm and gingival index score lower than 1. In all participants, plaque index (PI), gingival index (GI), probing depth (PD) and clinical attachment level (CAL) were measured at six sites around teeth^15^ and recorded by one calibrated periodontist.

All CP patients received non-surgical periodontal therapy. Oral hygiene instructions were given and scaling and root planing was performed within 10 days by one specialized physician (E.P.G.). Non-surgical periodontal therapy was performed under local anesthesia in four sessions, one quadrant in each session with site-specific gracey curettes (Hu-Friedy, Chicago, IL, USA). PI, GI, PD, CAL measurements were recorded and blood samples were taken at both the baseline and the sixth week of study.

### GCF sampling

Single-rooted teeth with moderate PD and CAL were chosen for GCF sampling in CP patients and equivalent teeth with healthy periodontium in HC group. GCF sampling was performed in proximal sites of the chosen tooth with a paper strip (ProFlow Inc., Amityville, NY, USA). The strip was placed into periodontal pocket in CP patients and gingival sulcus in HC group until a mild resistance was felt and held in place for 30 seconds^14^. Mechanical trauma and contamination of strip with saliva and/or blood were avoided. Contaminated strips were excluded and sampling was repeated 30 minutes later. All strips were placed into eppendorf tubes individually and kept at -80°C until ELISA analysis.

### Serum collection

To obtain serum samples, 5 mL of venous blood from antecubital vein were driven from each participant and placed into biochemical tubes containing an anticoagulant agent. Blood samples in tubes were allowed to rest for 30 minutes and then centrifuged at 6,000 rpm for 10 minutes. Serum was separated from tube and placed into eppendorf tubes. All serum samples were kept at -80°C until analysis.

### ELISA analysis

We added 100 μL of phosphate buffer solution into eppendorf tubes containing paper strips and mixed via vortex for a minute. IL-6 and IL-10 levels in GCF and serum samples were determined via ELISA kits (Invitrogen, Cambrillo, USA) according to the manufacturer's protocol. After the procedure, plates were read on a spectrometer at 450 nm wavelength. The results were converted to numeric values by using standard curves.

### DNA extraction and genotyping

The study protocol was carried out about our previous study^7^. For DNA extraction and genotyping, 2 mL of blood from the antecubital vein was driven from all participants and placed into biochemical tubes containing sodium EDTA. Tubes were then kept at -80°C until the genetic analysis day. A commercial kit (Invitrogen, Cambrillo, USA) was used for DNA extraction according to the manufacturer's instructions.

IL-10 gene polymorphism was evaluated via PCR-RFLP by analyzing IL-10 gene at position -597. Table 1 presents the primer sequences used for primer pairs.

**Table 1:** Clinical parameters at baseline and 6-week for study groups (mean ±SD)

		CP	C
PI	Baseline	1.9±0.6	
	6^th^ week	0.5±0.5^a^	0.1±0.3
GI	Baseline	2±0.3	
	6^th^ week	0.7±0.6^a^	0.1±1.3
PD	Baseline	5.4±0.6	
	6^th^ week	3±1^a^	2±0.7
CAL	Baseline	10±2.1	
	6^th^ week	8±2.1^a^	

a p<0.05

Amplification was performed via PCR (PTC-200 Thermal Cycler, MJ Research, Watertown, MA, USA) by using specific primers. PCR reaction mixtures (50 Nl) consisted of 5 Nl×10 PCR buffer, 0.2 mM each dNTP, 10 pmol of each primer (forward and reverse control and specific allele), 1.5 mM MgCl_2_, 200 ng of genomic DNA, 0.5 U Taq DNA polymerase (Fermantes, Maryland, USA) and internal control primers.

Afterwards, the amplification protocol was conducted as follows for IL-10 (-597) A/C: 1) Pre-PCR (94°C for 3 min), 2) PCR (35 cycles: 94°C for the 30 s, 48°C for 40 s, 72°C for 40 s); and 3) Elongation (72°C for 5 min). After these procedures, DNA was digested with restriction fragments and separated by size with the use of electrophoresis. RFLP amplification protocols were directed with the restriction enzyme RsaI. The fragments were also visualized on agarose gel at 3%. The expected results were at position -597, where A is present, leading to fragments of 42, 66, 232, and 240 bp in length, or 42, 232 and 306 bp, where C is present. In heterozygote individuals, the length of (A/C) were 42, 66, 232, 240 and 306 bp.

### Statistical evaluation

We performed statistical analysis using SPSS 15.0 (SPSS Inc., Chicago, IL, USA). Demographic data and clinical parameters were expressed as a mean±standard deviation. Genotype frequencies were defined by direct counting and allele frequencies were calculated from the observed number of genotypes. The frequencies of alleles and genotypes in patients with CP and controls were compared with the use of chi-square test and 95% confidence intervals. After the normality test, Mann-Whitney U and Wilcoxon tests were used. Gene polymorphisms were examined by logistic regression analysis while adjusting for potential confounding factors, including age and gender. The sample size was calculated for a significance level of 0.05 and the required sample size was 55 in the CP group, giving a statistical power of 80%^23^.

Genotype distributions of the groups and deviation of genotype frequencies from Hardy-Weinberg equilibrium were evaluated with chi-square test. Association was determined with odds ratios (ORs) and 95% confidence intervals. A p-value of <0.05 was considered statistically significant.

## Results

### Clinical parameters

Four participants from the CP group did not want periodontal treatment and were excluded from study. The CP group consisted of 17 men and 34 women (aged 20 to 46 years; mean age: 33.4±6.0). The healthy control group consisted of 17 men and 33 women (aged 23 to 46 years; mean age: 33±6.5). There was no significant difference in age and sex distributions in the groups (p>0.05).

Periodontal treatment decreased all clinical parameters in the CP group at the 6^th^ week compared to the baseline (p<0.05) (Table 1), but clinical parameters were still higher at the 6^th^ week compared to the HC group (p<0.05).

### Genotype distribution

IL-10 genotype frequencies of the groups showed disequilibrium according to Hardy-Weinberg (p<0.05). However, the allele frequencies of the IL-10 gene in study groups were in accordance with Hardy-Weinberg equilibrium (p>0.05).

IL-10 (-597) A/C genotype distribution was different between groups (p<0.05) (Table 2). In the CP group, IL-10 AA genotypes were associated to risk of periodontitis development 7 times higher in comparison with IL-10 AC/CC genotype with a statistically significant risk coefficient (OR:7, 95%CI, 2.83-60.25, p<0.05). The genotype distribution in the CP group was 35.3% for the AA genotype, 52.9% for the AC genotype, and 13.2% for the CC genotype. Also, allele frequencies were similar in the CP and healthy control groups (p>0.05).

**Table 2:** Allelic and genotypic frequencies observed in patients and healthy controls regarding the IL-10 (-597) G/C gene polymorphism. *p<0.05

	CP n=51	C n=50	P-value
IL-10(-597)			
CC	7(13.2)	3(6)	
AC	27(52.9)	45(90)	0.000*
AA	18(35.3)	2(4)	
**Allelic frequencies**			
IL-10(-597)			
C	39(38.2)	51(51)	0.089
A	63(61.7)	49(49)	

* p<0.05

### Biochemical parameters

At the 6^th^ week, IL-6 and IL-10 levels in GCF were lower than baseline in CP group (p<0.05) (Table 3). Both baseline and 6^th^ week levels of IL-6 in GCF of CP group were higher than those of the HC group (p<0.05). However, unlike GCF levels, serum levels of IL-6 and IL-10 in the CP group did not change after periodontal treatment (p>0.05). In the comparison between groups, serum cytokine levels were higher in the CP group than those of the control group (p<0.05). According to the ratio of IL-6/IL-10, serum and GCF IL-6/IL10 levels were significantly higher in the CP group than those of the control group (p<0.05). However, baseline and 6^th^ week values of IL-6/IL-10 ratio in GCF and serum were similar (p>0.05).

**Table 3:** Gingival crevicular fluid (GCF) and serum IL-6, IL-10 levels and IL-6/IL-10 ratio at baseline and 6-week (pg/30 s, mean ±SD)

			CP	C
	IL-6	Baseline	45.1±58.2^b^	
		6^th^ week	22.8±35.6^a,b^	14.5±28.1
GCF	IL-10	Baseline	17.8±28.1	
		6^th^ week	5.9±6.8^a^	6.9±13.3
	IL-6/IL-10	Baseline	3.6±5.9^b^	
		6^th^ week	4.6±8.3^b^	1.6±4.5
	IL-6	Baseline	23.9±63^b^	
		6^th^ week	23.7±61.5^b^	3.1±11.4
SERUM	IL-10	Baseline	3.3±6.8^b^	
		6^th^ week	3.7±8.9^b^	0.8±2.6
	IL-6/IL-10	Baseline	1.2±3.1^b^	
		6^th^ week	2±4.3^b^	0.5±2.9

We evaluated the changes in clinical and biochemical parameters in the CP group after categorizing patients according to IL-10 genotype distribution (IL-10 AA and AC+CC). Periodontal treatment provided significant improvements in both genotypes (p<0.05) (Table 4). However, clinical parameters between IL-10 AA and AC+CC genotypes were similar in both baseline and 6^th^ week (p>0.05) (Table 5).

**Table 4:** Gingival crevicular fluid (GCF) and serum IL-6, IL-10 levels and IL-6/IL-10 ratio at baseline and 6-week according to IL-10 genotypes (pg/30 s, Mean ±SD). a p<0.05 vs baseline

			GCF			SERUM	
		IL6	IL10	IL6/IL10	IL6	IL10	IL6/IL10
IL-10	Baseline	29,67±27.75	11.97±8.5	2.07±3.6	1.79±1.93	0.51±1.02	0.2±0.2
AA	6^th^ week	9.43±7.99	8.17±10.9	4.36±5.34	0.72±0.61	0.09±0.18	0.43±0.51
IL-10	Baseline	47.5±61.63	18.74±30	3.92±6.2	27.32±67.2	3.77±7.25	1.41±3.33
AC+CC	6^th^ week	24.95±37.9	5.65±6.29^a^	4.65±8.74	27.34±65.53	4.27±9.47	2.27±4.65

a p<0.05 vs. baseline

**Table 5:** Clinical parameters in chronic periodontitis (CP) group distributed by IL-10 genotypes (mean ±SD)

Clinical parameters	IL-10	
	AA	AC+CC
PI		
Baseline	1.75±0.5	1.9±0.6
6^th^ week	0.25±0.5	0.5±0.5
	P=0.01*	P=0.00*
GI		
Baseline	2±0	2±0.3
6^th^ week	0.75±0.9	0.7±0.5
	P=0.08	P=0.000*
PD		
Baseline	5.2±0.5	5.4±0.6
6^th^ week	3±0.8	3±1
	P=0.00*	P=0.000*
CAL		
Baseline	8.7±2.2	10.2±2
6^th^ week	7.2±1.5	8.1±2.2
	P=0.05*	P=0.000*

* p<0.05, vs. 6^th^ week

GCF IL-10 levels at the 6^th^ week in patients carrying IL-10 AC+CC genotype significantly decreased (p<0.05) (Table 4). In contrast, GCF and serum IL-6 levels in patients carrying the IL-10 AA genotype were not statistically different (p>0.05). Serum IL-10 levels in patients carrying IL-10 AA genotypes were not different from those of the IL-10 AC+CC genotypes at either baseline or 6^th^ week (p>0.05). The ratios of IL-6/IL-10, especially in serum, were lower in patients with IL-10 AA genotypes compared to IL-10 AC+CC genotypes (p<0.05).

Periodontal disease susceptibility in the CP group was evaluated with logistic regression analysis of IL-10 AA genotypes after adjustment of age and sex. Genotype diversity was not affected by either age or sex.

## Discussion

The complexity of cytokine network, together with gene polymorphisms of these cytokines, aids in uncovering the molecular mechanisms of inflammatory diseases, such as periodontal diseases. In this way, our study evaluated the role of anti-inflammatory (IL-10) and proinflammatory cytokines (IL-6) in chronic periodontitis patients. Correlation of these results with the gene polymorphism of IL-10 was also performed. As a result, we found that IL-10 AA genotypes were associated with chronic periodontitis and IL-10 AA genotype carriers showed lower IL-6/IL-10 levels in serum.

In this study, patients with the IL-10 AA genotype had a high susceptibility to chronic periodontitis. Similarly to our results, Sumer, et al.^12^ (2007) reported that the IL-10 gene polymorphism at position -597 seems to be associated with severe generalized CP. Association of SNPs in vascular endothelial growth factor (VEGF), IL-6, IL-10, IL-1beta, interferon alpha, TNF-alpha genes with immunoregulatory function revealed that C allele of VEGF, an allele of IL-10 and GG genotype of TNF-alpha, was individually associated with chronic periodontitis^10^. Although A alleles were higher in CP groups in this study, our results showed that the allele frequencies did not differ between chronic periodontitis and healthy control groups. However, Jaradat, et al.^14^ (2012) showed that the A positive allele genotypes (CA/AA) at position -597 seemed to increase the risk of chronic periodontitis. Unlike our results, Gonzales, et al.^8^ (2002) reported that IL-10 loci were not associated with either CP or AgP. Supporting Gonzales, et al.^13^ (2002), Reichert, et al.^22^ (2008) researched the German, Atanasovska-Stojanovska, et al.^23^ (2012), the Macedonian, and Brett, et al.^24^ (2005), the English Caucasians, and they found no association between CP patients and IL-10 -597 gene polymorphisms. Dissimilarity between the studied populations, number of subjects, differences in disease definition and experimental protocol (such as real-time PCR) might be the reason of dissimilarity among studies.

IL-10 is an important contributor to the pathogenesis of periodontal diseases^2^^,^^19^. Current results revealed that clinical outcomes were improved and IL-10 levels in GCF decreased after periodontal therapy. Similar to our results, several studies reported that serum IL-10 levels in patients with chronic periodontitis are higher than in periodontally healthy individuals^6^^,^^9^. In addition, the results of our study are different from the findings of Passoja, et al.^29^ (2010) which showed that the control group had significantly higher levels of IL-10 than the periodontitis groups.

IL-10 suppresses the production of IL-1, tumor necrosis factor- (TNF-)α, IL-6, IL- 8, IL-12, and IL-18. Also, similar to our results, IL-6 levels were elevated in periodontitis patients^16^^,^^17^ and decreased after successful periodontal therapy^4^^,^^5^. However, Reis, et al.^33^ (2014) reported that non-surgical periodontal therapy resulted in a decrease in IL-1, IL-1β, and IL-6 GCF levels, but not in IL-10 or TNF levels. In contrast, GCF IL-10 levels were associated with IL-6 levels in this study. Moreover, our results demonstrated that GCF and serum IL-6/IL-10 levels were higher in the CP group compared to healthy controls, which reflect local and systemic inflammation in periodontitis.

In addition, we evaluated whether the IL-10 genotypes could influence the levels of IL-6 and IL-10. IL-10 promoter gene polymorphisms have been associated with decreased serum IL-10 production^24^. However, IL-10 gene polymorphism and the amount of IL-10 depend on many factors, such as cell type, stimulation of time, kind of stimulus and the presence of other cytokines^25^. A recent study demonstrated that the A allele of IL-10-592 SNP was more prevalent in the CP group. It was also found that IL-10-592 SNP was functional and associated with lower levels of IL-10, TIMP-3 and OPG m-RNA expressions in diseased periodontal tissues^3^. Similarly, patients with AA genotypes presented lower levels of IL-10 and IL-6/IL-10 ratio in serum than those of the AC+CC genotype carriers in our study, but this reduction is not statistically significant. On the other hand, GCF IL-10 levels decreased after treatment in IL-10 AC+CC genotype carriers, but did not change in patients with AA genotypes.

In this study, we did not assess the haplotype analyses for this gene locus, which may be considered a shortcoming. Also, another limitation of this study is the low number of subjects included. However, we excluded periodontitis smoker patients because of the effects of smoking on the immune system.

## Conclusion

In conclusion, this study demonstrated that IL-10 -597 AA genotype appeared to be a risk factor for chronic periodontitis. Furthermore, genetic polymorphisms in the IL-10 gene might be useful as a marker to diagnose susceptibility to CP. On the other hand, an important finding of this study is that IL-10 AA genotype may tend to reduce IL-6 production or contribute to the inflammatory status, along with decreased GCF IL-10 levels. Nonetheless, further studies are needed to elucidate the mechanisms involved in cytokine gene polymorphisms and their production in periodontal disease.
